# Investigation of Cytotoxic and Apoptotic Effects of Styrax Liquidus Obtained From *Liquidambar orientalis* Miller (Hamamelidaceae) on HEp-2 Cancer Cell with Caspase Pathway

**DOI:** 10.5152/eurasianjmed.2023.23130

**Published:** 2023-10-01

**Authors:** Tuğçe Duran, Zeliha Tuncer

**Affiliations:** 1 Department of Medical Genetics, KTO Karatay University Faculty of Medicine, Konya, Turkey; 2 Department of Medical Biology, KTO Karatay University Faculty of Medicine Konya, Turkey; 3 Department of Translational Medicine, Ankara Yıldırım Beyazıt University Institute of Health Sciences, Ankara, Turkey

**Keywords:** *Liquidambar orientalis* Mill., styrax liquidus, caspase, apoptosis, HEp-2

## Abstract

**Objective:**

The HEp-2 cell line was first identified as laryngeal cancer cells. Then, it was reported to consist of cervical adenocarcinoma cells derived via HeLa cell line contamination. Styrax liquidus is an exudate that is provided by the injured hull of the *Liquidambar orientalis *Miller (Hamamelidaceae), which has been used for the treatment of skin problems, peptic ulcers, and parasitic infections or as an antiseptic. In our study, we purposed to research the cytotoxic and apoptotic effect of styrax liquidus on HEp-2 cancer cell line.

**Materials and Methods:**

The IC_50_ dosage of styrax liquidus (Turkish sweet gum obtained from trees) was set by the (3-(4,5-dimethylthiazol-2-yl)-2,5-diphenyltetrazolium bromide) assay, and the apoptotic effect of styrax liquidus on HEp-2 cancer cell was determined by assessing the expression of genes involved in apoptosis (*Bax*, *Bad*, *Bak1*, *p53*, *Bcl-2*, *Bcl-XL*, *Apaf-1*, *Caspase2*, *Caspase3a*, *Caspase9*, and *Caspase12*) by quantitative real-time polymerase chain reaction.

**Results:**

The IC_50_ value of styrax liquidus was found to be 125 μg/mL for 48 hours. According to the results, styrax liquidus reduced the population of HEp-2 laryngeal cancer cells and increased the expression of genes which were apoptosis related. These results indicate that styrax liquidus can be thought as a choice of cancer therapy.

**Conclusion:**

The finding of the study showed that it would be more useful to perform more qualified studies about the effect of styrax liquidus on cancer cells.

**Main Points**

- Styrax liquidus reduces the overall cell population with its cytotoxic effect on HEp-2 and HEK-293 cells.
- Styrax liquidus increases the expression of apoptosis-related genes.
- Styrax liquidus uses apoptotic caspase pathway to reduce cancer cell population.

## Introduction

In multicellular organisms, the proliferation, differentiation, and survival of cells are regulated according to the needs of the organism.^1^ In cancer cells, the mechanisms that regulate these events are disrupted, and the cells begin to divide uncontrollably. Despite the development of new treatment methods, cancer is still the leading cause of death in the world. According to 2021 GLOBOCAN (Global Cancer Observatory), there were approximately 10 million deaths in 2020.^2^ The increasing incidence of cancer has necessitated the creation of new approaches to cancer treatments.

Cell lines symbolize an important origin in science for cancer research. The HEp-2 cell line, first identified as laryngeal cancer cells in 1954 later, was announced to originate from cervical adenocarcinoma cells derived via HeLa cell line contamination in 1966.^3^

Cell death, which is a common feature of all living organisms, is a fundamental physiological process. It plays a role in many cellular processes such as embryonic development, aging, coordination of autoimmunity, and immunity.^4^ Apoptosis is the programmed death of cells that are aged, lost their function, developed irregularly or are damaged due to various reasons, and are no longer needed in the organism, under the control of special mechanisms.^5^ The proteins involved in the apoptotic pathway are called caspases.^6^ Caspases are called so for they have cysteine (c) residues in the catalytic sites and aspartate (asp) from the C-terminus of target proteins and act as enzymes.^7^ Numerous caspases have been identified in human cells, and these proteins are normally found in the cytoplasm of the cell in the form of inactive proenzymes. When the cell death signal arrives, pro caspases are converted to active caspases by a proteolytic process.^8^ Apoptosis culminates in the DNA breakdown into nucleosomal units, membrane blebbing, cell shrinkage, and the formation of apoptotic bodies.^9^ Apoptosis is mediated by 2 main pathways: cytochrome C-mediated cell death (intrinsic pathway) leading to the caspase activation cascade downstream of mitochondrial cytochrome C release, and cell death by the action of death signals (extrinsic pathway).^10^ In the extrinsic pathway, specific extracellular death receptors Fas cell surface death receptor or tumor necrosis factor receptor ligands bind at death receptors and lead to caspase activation.^11^ In the intrinsic pathway, apoptosis is regulated by modulated Bcl-2 and Bax proteins, as well as Apaf-1, and its balance is stimulated by the activation of caspase proteins (such as procaspase 9).^12^

**Figure 1.**
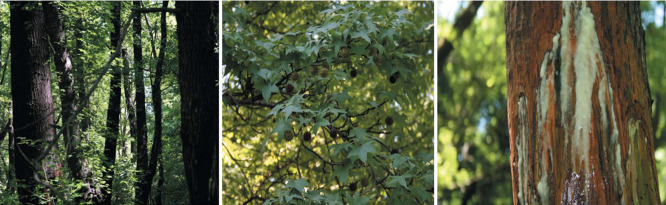
Anatolian sığla tree general external view and balsam appearance.^33^

Styrax liquidus (Turkish sweet gum) is an exudate taken from the injured trunk of the *Liquidambar orientalis *Mill. tree (Anatolian sığla tree) of the Hamamelidaceae family, Bucklandioidae subfamily.^13^ Turkish sweet gum is an endemic and relict plant species (it is a tertiary relict endemic taxon of the Eastern Mediterranean; it has adapted to different climatic changes by contributing to the floristic similarities from ancient times to the present).^14^ *L. orientalis*, which is mainly distributed in southwestern Turkey (Marmaris, Fethiye, Köyceğiz, Dalaman, and Ula districts),^13^ is a tertiary relict in similar climatic conditions in southern and western Anatolian regions of Turkey.^14^ Anatomical features of the Anatolian sığla tree are as follows: the sapwood is large and dirty reddish-white, the heartwood is pale reddish-brown, the aromatic smell of the oil can be easily felt, and it is delicate and quite uniform in terms of its satin shine wood. Normally, there are no balsam channels that secrete oil in the wood or bark of the Anatolian sığla tree. However, vertical balm channels, which are formed as a result of artificial or natural injury, occur in the annual rings in the direction parallel to the fibers, on the upper and lower sides of the wound (Figure 1).^13^

Styrax liquidus, which has survived from ancient times, was used for mummification in Egyptian civilization.^15^ Styrax liquidus, which started to be exported from southwestern Turkey, began to be used in medicine and cosmetics. Styrax liquidus has been used in the therapy of diverse diseases in Turkish Medicine. It has been used as an antiseptic in burns, wounds, peptic ulcers, and parasitic infections.^16,17^ It has been found that styrax liquidus has an anti-ulcer effect.^18^ In another study, it was shown that Turkish sweet gum oil has an antibacterial effect.^19^ The current study of Keyvan et al.^20^ reported that the styrax liquidus can be used to exert an antimicrobial effect on *Salmonella enteritidis *and *S. enteritidis *PT4. 

According to our knowledge, there are limited studies in the literature on the cytotoxic activity of styrax liquidus on cancer cells.^21,22^ In our study, we aimed to research the cytotoxic and apoptotic efficacy of styrax liquidus, an endemic plant derivative whose effects have not been investigated before, on HEp-2 laryngeal cancer and HEK-293 cells (Human Embryonic Kidney-293 cells) with apoptotic pathway and caspase pathway.

## Materials and Methods

This study was approved by KTO Karatay University Medical Faculty Non-pharmaceutical and Medical Device Research Ethics Committee (code: E.34335). All experiments were carried out in cell culture and molecular research laboratories within the KTO Karatay University Medical Faculty Central Research Laboratory.

Styrax liquidus was taken from local producers in Turkey and dissolved in DMSO (dimethyl sulfoxide) at certain concentrations. From the prepared master stock solution, HEp-2 and HEK-293 cells were applied with styrax liquidus at different molar concentrations and time intervals.

### Cell Culture

HEp-2 (CCL-23) cancer cell line and HEK-293 control cell line were obtained from American Type Culture Collection (Rockville, Md, USA). All cell lines used were seeded using sterile RPMI (Roswell Park Memorial Institute) 1640 medium containing 0.22 µM sterile-filtered 10% fetal bovine serum (FBS, Gibco, Thermo Fisher, Cleveland, OH, USA) and 1% penicillin/streptomycin antibiotic solution (Sigma-Aldrich, St. Louis, Mo, USA) and cultured. The culture conditions were 37°C in a humidified atmosphere of 5% CO_2_. After all cells were passaged into T75 flasks, with 80%-90% of the flask surface filled, they were harvested using 0.25% (w/v) trypsin–0.53 mM EDTA (Ethylene Diamine Tetraacetic Acid). After trypsinization, protease activity was inhibited with a fresh medium containing 10% FBS. After centrifugation at 1500 rpm for 5 minutes, the cell pellet was mixed with a new medium containing 1% DMSO and stored in cryovial tubes at –80°C.^23^

### Cytotoxicity Assay

To determine the IC_50_ (half maximal inhibitory concentration) dose of styrax liquidus, approximately 5 × 10^3^ cells/well were seeded into the 96 well-plate and cultured for 48 hours. Thereafter, the established doses (different molar concentrations) of styrax liquidus (7.5, 15, 31.25, 62.5, 125, 250, 500, and 1000 μg/mL) were applied to the wells, and the cells were cultured for 24-, 48-, and 72-hour time intervals. At least 5 wells were cultured in each dose group under the same conditions as the control group, with no styrax liquidus application. At 24, 48, and 72 hours after administration, cells in each well were treated with MTT (3-(4,5-dimethylthiazol-2-yl)-2,5-diphenyltetrazolium bromide) dye (Applichem, Darmstadt, Germany) dissolved in sterile 1× phosphate-buffered saline. Then, after approximately 3-4 hours, values of absorbance were measured at 570 nm in MultiskanSky microplate reader (Thermo Scientific). The percent cell viability was evaluated using the following formula:^24^



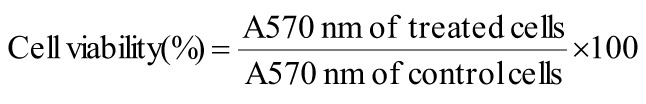



### Isolation of Total RNA and cDNA Synthesis

With the cytotoxic IC_50_ value of styrax liquidus, both control group cells and treated group cells were treated. Then, the cells were removed with trypsin–EDTA and centrifuged at 1500 rpm for 5 minutes. After gently mixing the cell pellet with TRIzol reagent (Sigma Aldrich®) for 5 minutes, it was subjected to phase separation with chloroform. RNA taken from the clear phase with isoamyl alcohol was precipitated at 13 000 g for 25 minutes at 4°C. In the final step, it was washed with 70% ethanol, and the RNA pellet was dried at room temperature. The RNA pellet was gently dissolved in DNase/RNase-free water (Ambion, Austin, Tex, USA). Different total RNA concentrations were equalized to 0.1 ng-5 μg after determination with the μdrop applicator on the MultiskanSky instrument (Thermo Scientific). cDNAs were synthesized using reverse transcriptase, oligo d(T), and random hexamer primers according to the Revertaid First Strand cDNA Synthesis Kit (Thermo Fisher Scientific) user instructions.^25^

### Quantitative Real-Time Polymerase Chain Reaction

Quantitative real-time polymerase chain reaction (qPCR) analysis was performed in triplicate with the QuantStudio™ 3 Real Time PCR System (Thermo Fisher Scientific).^26^ The nucleotide sequences of the primer pairs are shown in Table 1 as forward and reverse. To decide the state of apoptosis before and after styrax liquidus application; *Bax*, *Bad*, *Bak1*, *p53*, *Bcl-2*, *Bcl-XL*, *Apaf-1*, *Caspase2*, *Caspase3a*, *Caspase9*, and *Caspase12* gene expression levels were studied. Using the following thermal conditions, qPCR was performed for all samples and the indicated genes (Table 2).

### Statistical Analysis

For normalization, the *GAPDH *housekeeping/reference gene was used. Comparative Livak’s ΔΔCT method was used to calculate the relative gene expression.^27^ Cancer (styrax liquidus treated) and control (untreated) comparisons within groups (HEp-2 and HEK-293) were analyzed with the Statistical Package for the Social Sciences software, version 21 (IBM SPSS Corp.; Armonk, NY, USA) using the Student’s *t*-test. After normalization, ΔCt values were used in the analysis. The tests considered a baseline significance level of *P* < 0.05.

## Results

### Impacts of Styrax Liquidus on HEp-2 Cancer Cells and IC_50_ Value

**Figure 2.**
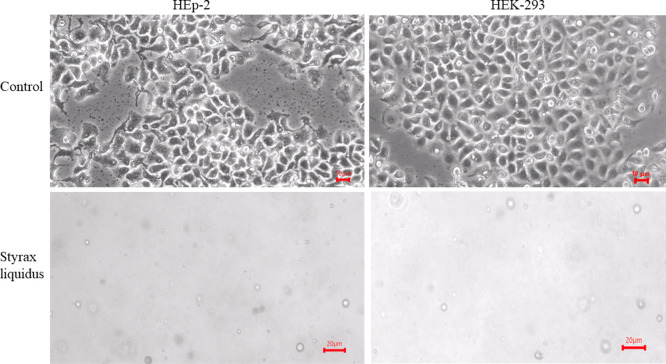
Morphological view of the cytotoxic effect of styrax liquidus on HEp-2 and HEK-293 cancer cells.

**Figure 3:**
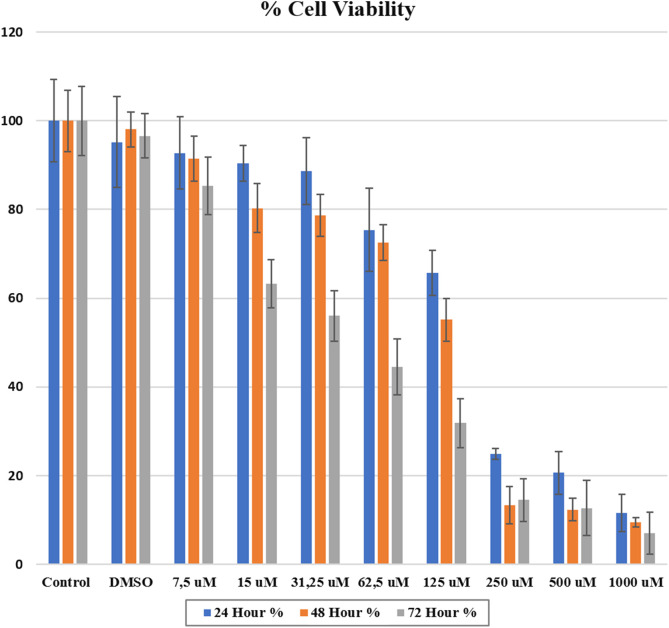
. The cytotoxic effect of styrax liquidus on HEp-2 cancer cell viability.

**Figure 4.**
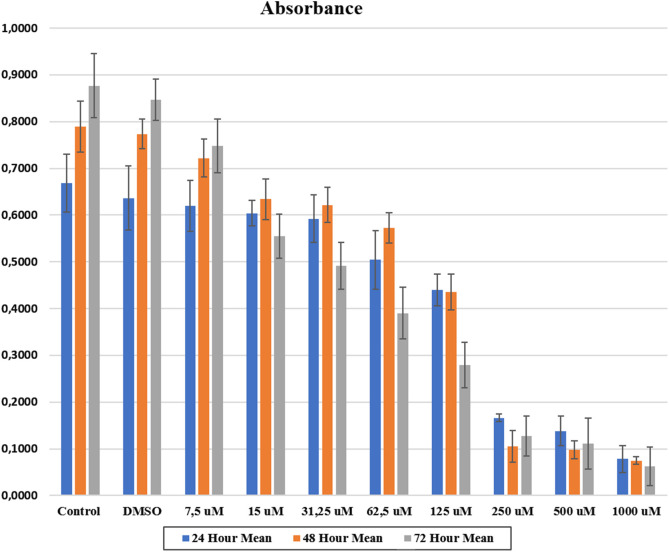
The cytotoxic effect of styrax liquidus on HEp-2 cancer cell absorbance values.

Impacts of styrax liquidus on HEp-2 cancer cells determined by the application of different concentrations of styrax liquidus (7.5, 15, 31.25, 62.5, 125, 250, 500, and 1000 μg/mL) to cultured HEp-2 cancer cells for 24-, 48-, and 72-hour intervals. According to MTT cell viability assay, the IC_50_ value of styrax liquidus was determined to be 125 μg/mL for 48 hours. Results indicate that styrax liquidus has a highly cytotoxic effect on HEp-2 cancer cells and HEK-293 control cells (Figure 2). Cell viability and absorbance are shown in Figures 3 and 4, respectively.

### Apoptotic and Caspase Pathway Quantitative Polymerase Chain Reaction Results of HEp-2 and HEK-293 Cells

**Figure 5.**
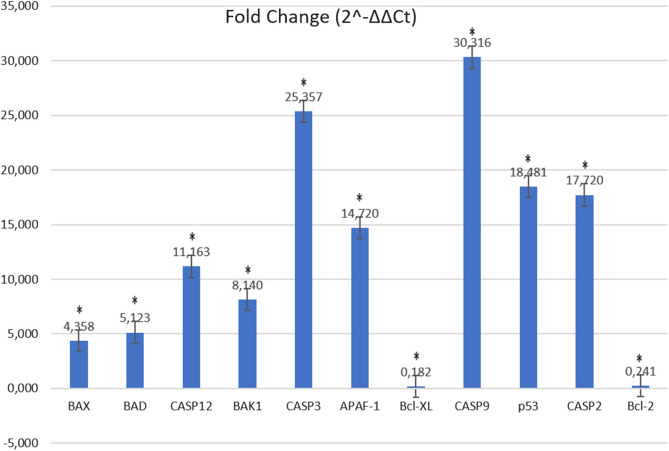
Fold-change graph of the result of gene expression analyses of apoptosis-related and caspase pathway genes in HEp-2 cell line.

**Figure 6.**
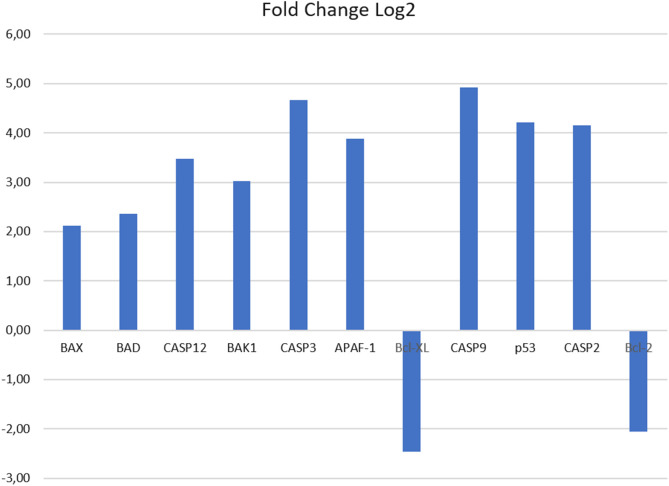
Fold-change log2 graph of the result of gene expression analyses of apoptosis-related and caspase pathway genes in HEp-2 cell line.

**Figure 7.**
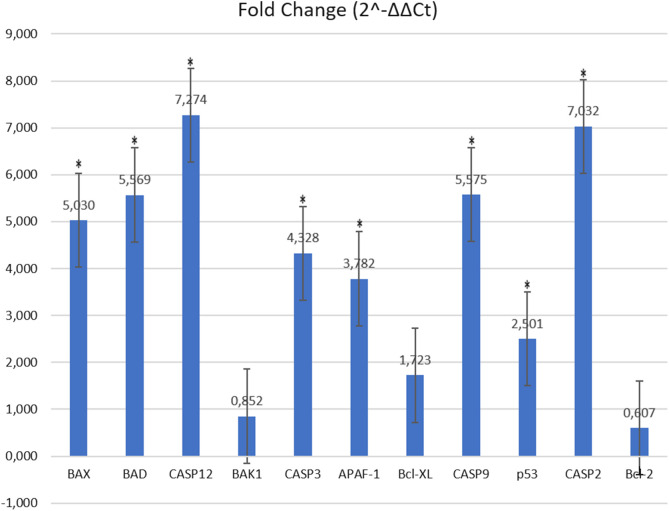
Fold-change graph of the result of gene expression analyses of apoptosis-related and caspase pathway genes in HEK-293 cell line.

**Figure 8.**
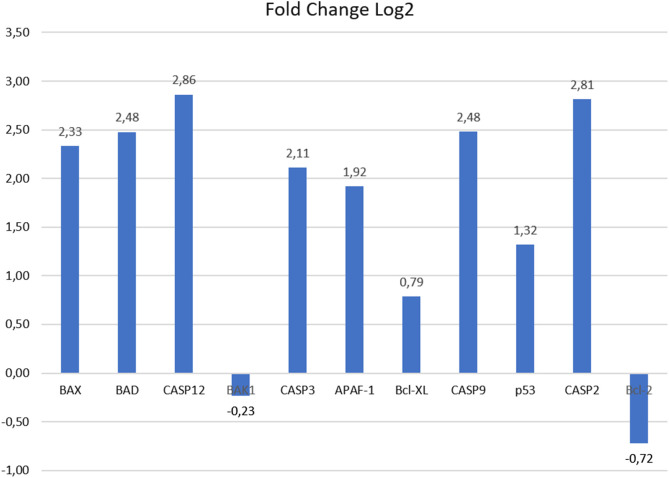
Fold-change log2 graph of the result of gene expression analyses of apoptosis-related and caspase pathway genes in HEK-293 cell line.

To evaluate the impact of styrax liquidus on the apoptosis and caspase pathway, apoptosis-related expression levels of the genes *Bax*, *Bad*, *Bak1*, *p53*, *Bcl-2*, *Bcl-XL*, *Apaf-1*, *Caspase2*, *Caspase3a*, *Caspase9*, and *Caspase12 *were determined in HEp-2 cancer cells. Relative gene expression levels were assessed after the normalization with *GAPDH *housekeeping gene expression. According to the qPCR results, styrax liquidus administration increased the expression of *Bax*, *Bad*, *Bak1*, *p53*, *Apaf-1*, *Caspase2*, *Caspase3a*, *Caspase9*, and *Caspase12 *and decreased the expression of *Bcl-2 *and* Bcl-XL* in HEp-2 cancer cells (*P *< 0.05). For HEK-293 cancer cells; *Bax*, *Bad*, *p53*, *Apaf-1*, *Caspase2*, *Caspase3a*, *Caspase9*, *Caspase12*, and* Bcl-XL *gene expression increased (*P* < 0.05). *Bak1 and Bcl-2* gene expression decreased, and this difference was not significant (*P *> 0.05). A statistically significant difference was found between the expression of all genes (*Bax*, *Bad, Bak1*, *p53*, *Apaf-1*, *Caspase2*, *Caspase3a*, *Caspase9*, *Caspase12*, *Bcl-2*, and *Bcl-XL*) for the HEp-2 cell line (*P* < 0.05). In Figures 5 and 6, the fold change (2^–ΔΔCT^) and fold-change log2 graphs of the expression profile changes of the apoptotic marker genes on HEp-2 cells are shown, whereas in Figures 7 and 8 the fold change (2^–ΔΔCT^) and fold-change log2 graphs of the expression profile changes of the apoptotic marker genes on HEK-293 cells are shown, respectively.

## Discussion

Cancer is a disease of genetic origin, which is associated with many signaling pathways and is defined by aberrant differentiation and uncontrolled proliferation of cells. Despite the increase in treatment options for cancer and advances in technology, the incidence of cancer is increasing every year. Considering all the diseases that cause death, cancer has been reported as the most common cause of death after cardiovascular diseases.^28,29^ The increasing incidence of cancer worldwide has necessitated the creation of new approaches to the effective treatment of cancer. Cell lines are in vitro model systems that are widely used in basic cancer research. The HEp-2 cell line that identified as laryngeal cancer cells was reported then to be included in cervical adenocarcinoma cells derived by HeLa cell line contamination.^30^ Styrax liquidus (Turkish sweet gum) obtained from *L. orientalis* Mill., an endemic plant that has been used in Turkish folk medicine for centuries, is known as a resinous exudate and is used in traditional treatment approaches.^31^ In our study, the apoptotic and cytotoxic effects of styrax liquidus on the HEp-2 cell line were investigated through apoptosis pathway elements and caspase pathway; as a result, the cytotoxic effect of styrax liquidus on HEp-2 cells was demonstrated by the MTT assay at 125 µM for 48 hours.

There are a few studies investigating the cytotoxic effect of styrax liquidus on cancer cell lines. According to Atmaca et al.^21^, *L. orientalis* Mill. reported that plant extract induces autophagy in prostate cancer via the cell cycle regulator PI3K/AKT/mTor intracellular signaling pathway. They used prostate PC-3 and DU-145 cancer cell lines to research the styrax liquidus impact on cell viability by XTT assay. They reported that styrax liquidus IC_50_ values were 37.8 μg/mL and 25.9 μg/mL in PC-3 and DU-145 cells at 72 h, respectively. In another study, styrax liquidus IC_50_ value was found to be 50.22 μg/mL at 24 hours on L929 cancer cell line by the MTT assay.^32^ It is thought that the reason for the IC_50_ value to be different in the studies may be because of the different cell lines and cytotoxicity tests used, even laboratory conditions. There is a need for more cytotoxicity studies investigating the efficacy of styrax liquidus in cancer cell lines.

According to the result of this study, after the application of styrax liquidus HEp-2 and HEK-293 cells, *Bax*, *Bad*, *p53*, *Apaf-1*, *Caspase2*, *Caspase3a*, *Caspase9*, and *Caspase12 *genes expression increased; this result suggested that decrease in proliferation was caused by the apoptosis pathway. In addition to the apoptosis-signaling pathway, cell cycle checkpoints and autophagy pathways can also be studied in further studies.

It is necessary to mention an important limitation of this study that can be addressed in future research. Cancer and control cells were studied. It was observed that styrax liquidus had a toxic effect on healthy cells as well as on cancer cells, as in classical chemotherapy treatments. Current cancer treatments and smart drug strategies have taken an approach that targets only cancer cells without harming healthy cells. However, since there are very few studies on styrax liquidus cancer cells, the study was carried out to contribute to the literature. We noticed that there are very few cancer studies on styrax liquidus, and it is planned to investigate its effectiveness in other cancer cells in future studies.

**Figure 9.**
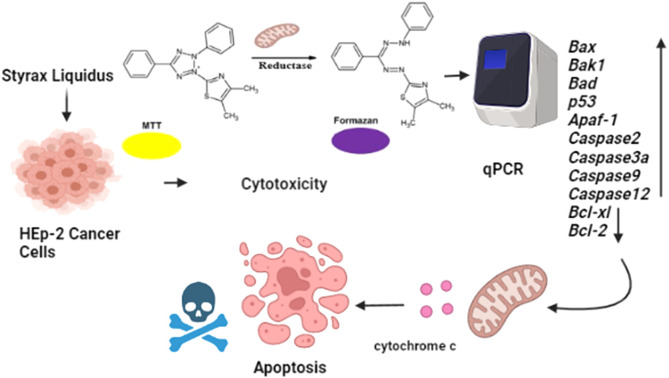
Graphical abstract showing the apoptotic effect of styrax liquidus on HEp-2 cancer cells.

In conclusion, we found that the application of styrax liquidus reduced the overall cell population in HEp-2 and HEK-293 cells and showed a cytotoxic effect. Styrax liquidus increases (up-regulaion) the apoptosis-related gene expression; this result shows that styrax liquidus use apoptotic and caspase pathways (Figure 9). The use of styrax liquidus is effective in preventing the proliferation of cancer cells. The result of this study may contribute to styrax liquidus serving as a potential drug candidate for the treatment of cervical or laryngeal cancers, as well as different combination drug therapies. In the future, detailed therapeutic and pharmacogenetic studies may be needed for different cancer treatment approaches.

## Figures and Tables

**Table 1.** Forward and Reverse Nucleotide Sequences of All Primers Used in the Real-time PCR Stage

**Gene**	**Oligonucleotide Sequence (5ʹ-3ʹ)**	**Amplicon Size**
*Bax*	F-CCCGAGAGGTCTTTTTCCGAG	155
	R-CCAGCCCATGATGGTTCTGAT	
*Bad*	F-CCCAGAGTTTGAGCCGAGTG	249
	R-CCCATCCCTTCGTCGTCCT	
*Bak1*	F-CATCAACCGACGCTATGACTC	192
	R-GTCAGGCCATGCTGGTAGAC	
*p53*	F-CAGCACATGACGGAGGTTGT	125
	R-TCATCCAAATACTCCACACGC	
*Bcl-2*	F-GGTGGGGTCATGTGTGTGG	89
	R-CGGTTCAGGTACTCAGTCATCC	
*Bcl-XL*	F-GAGCTGGTGGTTGACTTTCTC	119
	R-TCCATCTCCGATTCAGTCCCT	
*APAF-1*	F-AAGGTGGAGTACCACAGAGG	116
	R-TCCATGTATGGTGACCCAT	
*Casp2*	F-AGCTGTTGTTGAGCGAATTGT	124
	R-AGCAAGTTGAGGAGTTCCACA	
*Casp3*	F-CATGGAAGCGAATCAATGGACT	139
	R-CTGTACCAGACCGAGATGTCA	
*Casp9*	F-CTCAGACCAGAGATTCGCAAAC	116
	R-GCATTTCCCCTCAAACTCTCAA	
*Casp12*	F-AACAACCGTAACTGCCAGAGT	118
	R-CTGCACCGGCTTTTCCACT	
*GAPDH*	F-GGAGCGAGATCCCTCCAAAAT	197
	R-GGCTGTTGTCATACTTCTCAT	

PCR, polymerase chain reaction.

**Table 2.** qPCR Conditions Used in Gene Expression Analysis

**Stage**	**Temperature (°C)**	**Time**	**Cycle**
Pre-denaturation	95	5 minutes	1×
Denaturation	95	10 seconds	40×
Annealing	56-60	20 seconds	
Extension	72	60 seconds	
Cooling	4	∞	–

qPCR, quantitative polymerase chain reaction.
